# The tumor immune microenvironment and immune-related signature predict the chemotherapy response in patients with osteosarcoma

**DOI:** 10.1186/s12885-021-08328-z

**Published:** 2021-05-21

**Authors:** Lijiang He, Hainan Yang, Jingshan Huang

**Affiliations:** 1 Department of Orthopaedics, The Second Affiliated Hospital of Fujian Medical University, Quanzhou, Fujian China; 2 Department of Ultrasound, The Second Affiliated Hospital of Fujian Medical University, Quanzhou, Fujian China; 3 Department of General Surgery, The Second Affiliated Hospital of Fujian Medical University, Quanzhou, 362000 Fujian China

**Keywords:** Osteosarcoma, Immune-related gene, Tumor immune microenvironment, Predictive signature, Chemotherapy

## Abstract

**Background:**

Genome-wide expression profiles have been shown to predict the response to chemotherapy. The purpose of this study was to develop a novel predictive signature for chemotherapy in patients with osteosarcoma.

**Methods:**

We analysed the relevance of immune cell infiltration and gene expression profiles of the tumor samples of good responders with those of poor responders from the TARGET and GEO databases. Immune cell infiltration was evaluated using a single-sample gene set enrichment analysis (ssGSEA) and the CIBERSORT algorithm between good and poor chemotherapy responders. Differentially expressed genes were identified based on the chemotherapy response. LASSO regression and binary logistic regression analyses were applied to select the differentially expressed immune-related genes (IRGs) and developed a predictive signature in the training cohort. A receiver operating characteristic (ROC) curve analysis was employed to assess and validate the predictive accuracy of the predictive signature in the validation cohort.

**Results:**

The analysis of immune infiltration showed a positive relationship between high-level immune infiltration and good responders, and T follicular helper cells and CD8 T cells were significantly more abundant in good responders with osteosarcoma. Two hundred eighteen differentially expressed genes were detected between good and poor responders, and a five IRGs panel comprising TNFRSF9, CD70, EGFR, PDGFD and S100A6 was determined to show predictive power for the chemotherapy response. A chemotherapy-associated predictive signature was developed based on these five IRGs. The accuracy of the predictive signature was 0.832 for the training cohort and 0.720 for the validation cohort according to ROC analysis.

**Conclusions:**

The novel predictive signature constructed with five IRGs can be effectively utilized to predict chemotherapy responsiveness and help improve the efficacy of chemotherapy in patients with osteosarcoma.

**Supplementary Information:**

The online version contains supplementary material available at 10.1186/s12885-021-08328-z.

## Background

Primary bone cancer is a rare malignant tumor that originates in the bones and accounts for far less than 1% of all cancers [1, 2]. Osteosarcoma is the most frequent malignancy of primary bone cancer and commonly occurs in children and teenagers [3]. Currently, comprehensive treatment strategies for osteosarcoma include surgery, chemotherapy, radiotherapy and targeted therapy [4]. Most often, chemotherapy is the most frequently used strategy for patients with osteosarcoma before surgery. Neoadjuvant chemotherapy shrinks the tumor and makes surgery easier in patients with osteosarcoma [5]. Before the introduction of chemotherapy, approximately 90% of patients with osteosarcoma developed distant metastases, which is the leading cause of death for patients with osteosarcoma [6]. The advent of chemotherapy has dramatically improved the 5-year overall survival (OS) rate from less than 20 to 60% in patients with osteosarcoma [7]. However, therapy resistance and chemoinsensitivity remain major challenges for osteosarcoma treatment. Despite numerous clinical trials attempting to improve the outcomes of poor responders by modifying chemotherapy regimens and intensifying postoperative therapy, their prognosis remains poor [8–10].

The response to chemotherapy significantly influences the incidence of local recurrence and prognosis of osteosarcoma [11]. Patients with osteosarcoma who have a good response to chemotherapy represent a subset of patients with osteosarcomas exhibiting innate sensitivity to treatment, resulting in a superior response and good prognosis. Patients who display a poor response to chemotherapy have a much higher risk of recurrence and poor outcomes even after complete resection of the primary tumor [12]. Tumor necrosis induced by chemotherapy remains the only reliable method to assess the effects of chemotherapy [13]. However, the tumor necrosis rate is only able to be detected in surgically resected specimens, and histological response monitoring during the course of chemotherapy treatment is impossible. Thus, developing a predictive signature that discriminates patients who will respond to chemotherapy before treatment may help to improve the efficacy of chemotherapy in patients with osteosarcoma.

Recently, with the development of deep sequencing techniques and gene microarrays, bioinformatic analyses of genome-wide expression profiles have been broadly performed to distinguish patients who will or will not respond to chemotherapy [14–16]. Currently, multigene signatures for the prediction of the chemotherapy response have been widely employed using gene expression analysis with gene microarrays or deep sequencing techniques [17]. Here, we strived to develop a predictive model based on gene expression profiles for predicting the response to chemotherapy in patients with osteosarcoma. The tumor immune microenvironment (TIME) plays a critical role in regulating tumor progression and the response to chemotherapy [18]. An increasing number of studies have reported that a higher level of intratumor immune cell infiltration is associated with a better response to chemotherapy [19–21]. Therefore, we reasoned that a predictive signature based on IRGs would likely be more strongly predictive of chemotherapy responsiveness in patients with osteosarcoma.

In this study, we attempt to construct a signature with the capability to predict the chemotherapy response and prevent patients who are not sensitive to chemotherapy from receiving ineffective treatment. In this study, we systematically surveyed tumor-infiltrating immune profiles between good responders and poor responders employing CIBERSORT and ssGSEA using data from the TARGET and GEO databases. We explored the predictive value of IRGs for the chemotherapy response. Finally, we established a novel immune gene-based signature that may be clinically useful for predicting responsiveness to chemotherapy in patients with osteosarcoma. Furthermore, we aimed to obtain a deeper understanding of the underlying mechanism of chemosensitivity and chemoresistance that could lead to the development of personalized medicine for patients with osteosarcoma.

## Methods

### Data acquisition and preprocessing

The Therapeutically Applicable Research to Generate Effective Treatments (TARGET) program applies a comprehensive genomic approach to determine molecular changes that drive paediatric cancers. The goal of TARGET data is to facilitate the discovery of therapeutic targets for paediatric cancers and facilitate the rapid translation of those findings into clinical applications. Gene Expression Omnibus (GEO) is a public database repository of high-throughput gene expression data and other functional genomics datasets.

The patients who fulfilled the following inclusion criteria were selected from the TARGET and GEO databases: 1) gene expression was detected using a microarray or high-throughput sequencing, and 2) the histological response to chemotherapy was recorded. One hundred thirty-seven patients who fulfilled the abovementioned criteria were selected from four datasets (TARGET, GSE14827, GSE87437 and GSE39055). The TARGET cohort was used as the training dataset to construct the signature for the prediction of chemotherapy response. The GSE14827 and GSE87437 cohorts served as validation datasets. The GSE14827 and GSE87437 cohorts were annotated according to the Affymetrix Human Genome U133 Plus 2.0 Array platforms. The “sva” R package containing the “Combat” function was applied to remove batch effects. Clinical information of patients with osteosarcoma was obtained from the TARGET and GSE39055 cohorts.

### Histological evaluation of the chemotherapy response

The chemotherapy response was assessed by comparing tumor necrosis in the biopsy specimens or surgically resected specimens. Tumor necrosis was graded as follows: grade IV (100% necrosis), grade III (90–99% necrosis), grade II (50–89% necrosis), and grade I (0–49% necrosis). In this study, patients with a tumor necrosis rate less than 90% were defined as poor responders, and patients showing a tumor necrosis rate of 90% or greater were defined as good responders.

### Evaluation of tumor-infiltrating immune cells and the immune infiltration level

The CIBERSORT algorithm was used to quantify the proportions of immune cells in a mixed cell population in both the training and validation cohorts [22]. The normalized gene expression data were analysed to calculate the abundance ratio matrix of 22 immune cell types in each sample, including macrophages (M1 macrophages, M2 macrophages, and M0 macrophages), T cell types (T follicular helper (Tfh) cells, resting memory CD4 T cells, activated memory CD4 T cells, γδ T cells, CD8 T cells, Tregs, and naïve CD4 T cells), resting natural killer cells, activated NK cells, resting mast cells, activated mast cells, memory B cells, resting dendritic cells, activated DCs, naïve B cells, monocytes, plasma cells, neutrophils and eosinophils [23]. The CIBERSORT results of samples with *p* < 0.05 indicated that the inferred fractions of immune cell populations were accurate and were eligible for further analysis. The CIBERSORT output estimates were normalized, and immune cell type fractions were summed to one. Spearman’s correlation analysis was performed to identify the relationship of each immune cell type.

Single-sample gene set enrichment analysis (ssGSEA) was applied to quantify the enrichment levels of the 29 immune signatures in each osteosarcoma sample [24]. Next, the ESTIMATE algorithm was used to calculate the immune cell infiltration level (immune score) for each osteosarcoma sample with the “ESTIMATE” R package [25]. In addition, patients with osteosarcoma were divided into high and low immune infiltration subtypes based on the median value of immune scores.

### Identification of differentially expressed genes and functional enrichment analyses

The differentially expressed genes between good responders and poor responders were screened using the “limma” R package. A *p* value < 0.05 and | log FC| ⩾ 0.5 were set as the cut-off criteria.

Functional enrichment analyses were performed to investigate the possible molecular mechanisms of differentially expressed genes using the “clusterProfiler” package. Terms identified in GO and KEGG analyses with a false discovery rate (FDR) < 0.05 were considered statistically significant and were visualized using the “digest” and “GOplot” packages.

### Construction of an immune-related signature for the prediction of the chemotherapy response

The comprehensive list of IRGs was downloaded from the Immunology Database and Analysis Portal (ImmPort) database (https://immport.niaid.nih.gov), which shares immunology data and provides a list of IRGs for cancer researchers.

Least absolute shrinkage and selection operator (LASSO) logistic regression analysis was used to identify and select the optimal genes in the training cohort with the “glmnet” R package. The parameter λ selection in the LASSO model was tuned using ten-fold cross-validation. Next, a binary logistic regression analysis was carried out to discover the genes with predictive value for the chemotherapy response, and the selected genes with a nonzero coefficient were combined into a gene signature. Each gene in the signature has a regression coefficient (β) for predicting chemotherapy response that was calculated as follows: index = (expression of gene 1)*β1 + (expression of gene 2)*β2 + (expression of gene 3)*β3...… + (expression of gene n)*βn, where n indicates the number of the gene.

Principal component analysis (PCA) was used to examine the distribution of samples. The PCA plot was drawn across the first two principal components. A receiver operating characteristic (ROC) curve was generated to obtain the area under the curve (AUC) accuracy and sensitivity in the training and validation cohorts using the “pROC” R package and to evaluate the discriminative power of the signature.

### Gene set enrichment analysis

We applied a gene set enrichment analysis (GSEA) between the good response and poor response groups separated by the immune-related predictive signature using clusterProfiler and the enrichplot R package. Two functions (gseGO and gseKEGG) were applied to identify the enriched terms in Gene Ontology (GO) and Kyoto Encyclopedia of Genes and Genomes (KEGG) databases, and *p* < 0.05 and FDR < 0.25 were considered statistically significant in the GSEA.

### Statistical analysis

In this study, all statistical analyses were conducted using R software (version 3.6.5). Continuous variables were compared using Student’s t-tests. Survival analyses were conducted using the Kaplan–Meier method with the log-rank test by the “survival” R package. All statistical analyses were two-sided, and statistical significance was defined at *p* values < 0.05. For the hierarchical clustering analysis, Pearson’s correlation coefficients and the averaging method were used, and the results were shown in heat-maps.

## Results

### Characteristics of the cohorts

First, 137 patients from the TARGET, GSE14827 [26], GSE87437 [27] and GSE39055 [28] databases were selected. The training cohort consisted of 52 patients from TARGET, including 21 good responders and 31 poor responders. The validation cohort was composed of the combination of 27 patients from GSE14827 and 21 patients from GSE87437, including 21 good responders and 27 poor responders. The GSE39055 dataset consisted of 37 patients, including 14 good responders and 23 poor responders. Detailed information is available for the TARGET and GSE39055 cohorts, including OS data. No significant differences in the clinical characteristics were observed between the training and validation cohorts stratified into good responders or poor responders (Table 1).

**Table 1:** Clinicopathological characteristics of patients with osteosarcoma

	Training cohort		Validation cohort GSE14827(*n* = 27) and GSE87437(*n* = 21)			
	TARGET(*n* = 52)				GSE39055(*n* = 37)	
Response	Good (*n* = 21)	Poor (*n* = 31)	Good (*n* = 21)	Poor (*n* = 27)	Good (*n* = 14)	Poor (*n* = 23)
**Age (years)**						
Median	15	14	15	15	11	12
Range	9 to 32	9 to 39	5 to 38	8 to 24	4 to 23	4 to 71
**Gender**						
Female	8	13	9	7	6	12
Male	13	18	12	20	8	11
**Recurrence**						
Yes	6	19	–	–	4	14
No	15	12	–	–	10	9
**Survival (months)**						
Median	52.7	35.4			65.15	29
Range	18 to 131	9 to 194			14 to 200.9	2.7 to 196.1
**Status**						
Alive	17	14	–	–	14	14
Dead	4	17	–	–	0	9

**Fig. 1:**
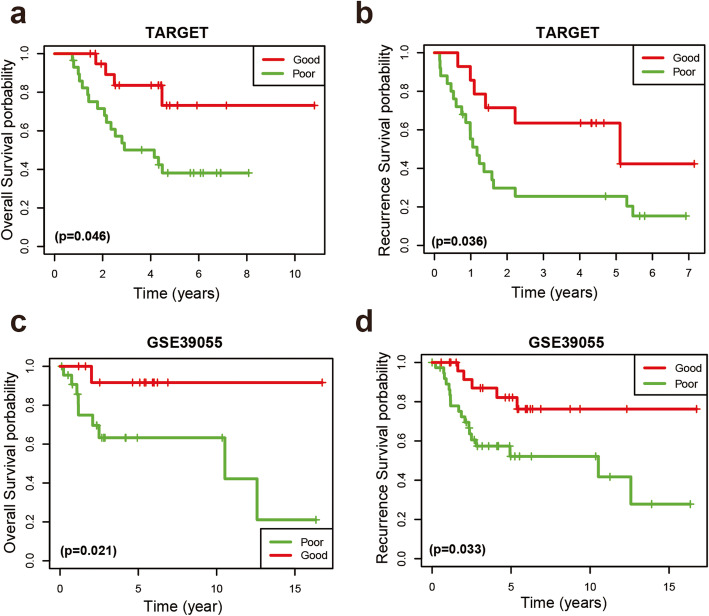
Kaplan–Meier curves for OS and RFS in patients with osteosarcoma. **a, b** Kaplan–Meier survival curves depicting OS and RFS in patients with good response and poor response in the TARGET cohort. **c, d** Kaplan–Meier survival curves depicting OS and RFS in patients with good response and poor response in the GSE39055 cohort

According to the Kaplan-Meier survival analysis, the OS of the good responder group was better than that of the poor responder group in both the TARGET and GSE39055 cohorts (Fig. 1a and c). The 5-year OS rate was 73.1% for good responders and 38.1% for poor responders in the TARGET cohort. Furthermore, we observed a positive relationship between the chemotherapy response and recurrence-free survival rate (RFS) in both the TARGET and GSE39055 cohorts (Fig. 1b and d). Univariate and multivariate Cox proportional hazards regression analyses were performed on the TARGET and GSE39055 cohorts to further analyse the correlations between OS, chemotherapy response, age, and sex. The chemotherapy response served as an independent prognostic factor in the TARGET (Table S1) and GSE39055 (Table S2) cohorts in both univariate and multivariate analyses. These results indicated that the response to chemotherapy is a significant prognostic factor for patients with osteosarcoma.

### Immune cell infiltration was associated with the chemotherapy response in patients with osteosarcoma

**Fig. 2:**
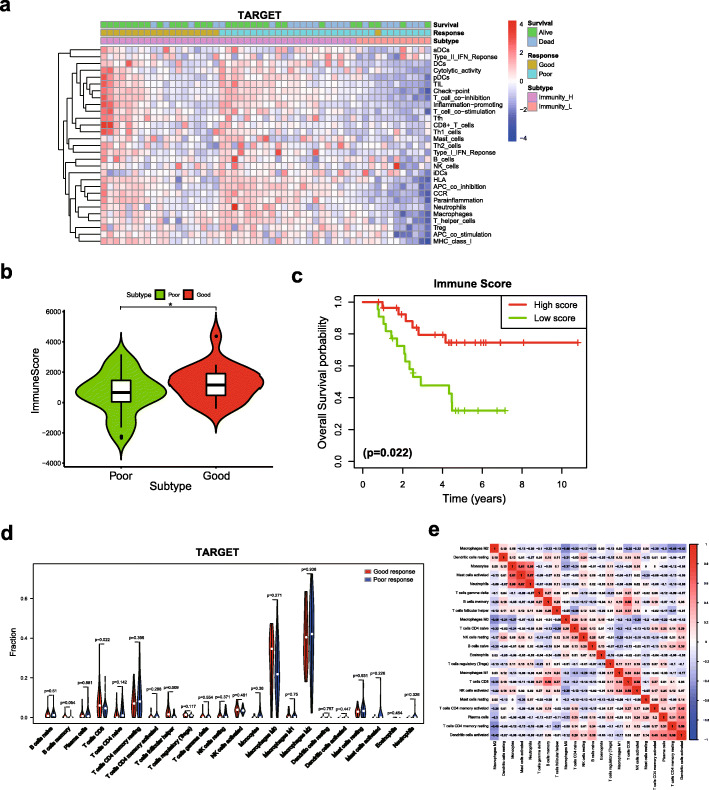
The relationship between immune cell infiltration and the chemotherapy response. **a** Unsupervised clustering analysis of patients with osteosarcoma who achieved good and poor responses from the TARGET cohort using ssGSEA. **b** Comparison of the immune scores between good responders and poor responders in the TARGET cohort. **c** Kaplan-Meier curves for OS showing that the high immune cell infiltration subtype had a favourable outcome compared with the low immune cell infiltration subtype. **d** Violin plot of good responders and poor responders in the TARGET cohort. **e** Correlation matrix of 22 immune cell type proportions in the TARGET cohort. * *p* < 0.05

Several studies have revealed the close association between the chemotherapy response and the tumor immune microenvironment [19–21]. A ssGSEA was used to assess the immune infiltration level between good responders and poor responders and to explore the potential correlation between the chemotherapy response and immune infiltration in patients with osteosarcoma. Patients in the TARGET cohort were divided into high and low immune cell infiltration subtypes based on the immune score. Our results clearly showed that most good responders belonged to the high-level immune infiltration subtype (Fig. 2a), and good responders had a higher immune score than poor responders (Fig. 2b). In addition, Kaplan-Meier curves revealed that the high-level immune cell infiltration subtype was significantly associated with an improved prognosis for patients with osteosarcoma (Fig. 2c). We then performed the same analyses on the validation cohort and GSE39055 cohort. Similar results were observed in both cohorts, and good responders were significantly related to high-level immune infiltration (Fig. S1a, b).

We utilized the CIBERSORT algorithm to calculate the percentages of 22 immune cell types between good responders and poor responders in the TARGET cohort and to further determine the relationship between immune cells and the chemotherapy response. Our results showed that M0 macrophages and M2 macrophages accounted for a large proportion of osteosarcoma immune cell subsets. T follicular helper (Tfh) cells and CD8 T cells were significantly more abundant in good responders (Fig. 2d). Among these 22 immune cell types, CD8 T cells exhibited the strongest positive correlation with activated NK cells (Pearson’s correlation coefficient = 0.58) but showed a negative correlation with M2 macrophages (Pearson’s correlation coefficient = − 0.35) (Fig. 2e). Similar results were observed in the GSE39055 and GSE14827 cohorts, as the percentages of CD8 T cells and Tfh cells were increased in the good responder group (Fig. S2a, b). Taken together, the results presented above supported a positive association between immune cell infiltration and the chemotherapy response.

### Identification of differentially expressed genes between good responders and poor responders

**Fig. 3:**
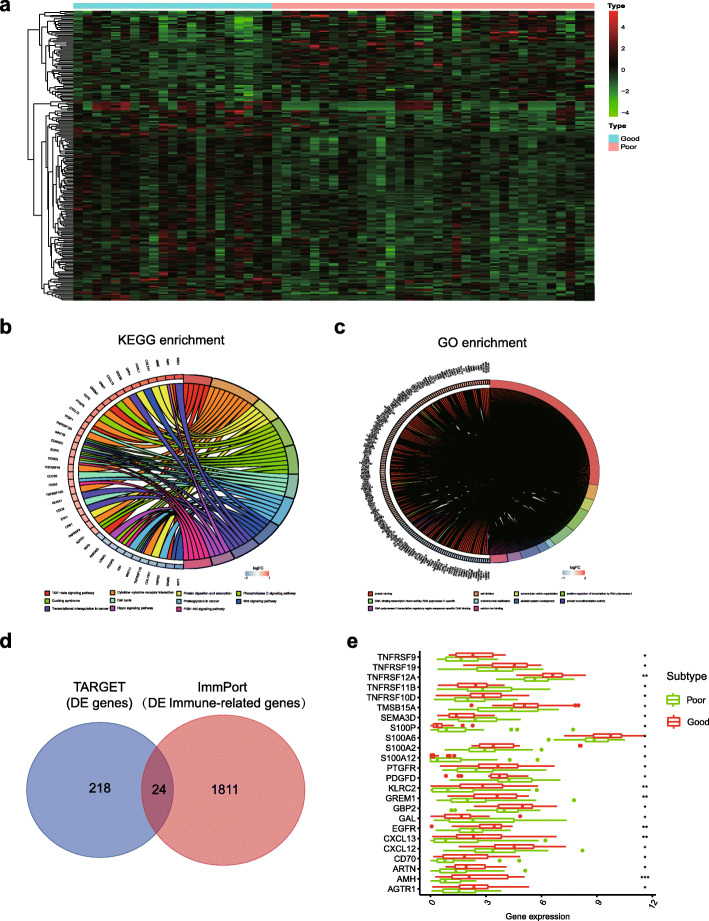
Differentially expressed genes and functional enrichment analyses. **a** Heatmap of 218 differentially expressed genes between good responders and poor responders. **b** The top 10 significant pathways identified in the KEGG enrichment analysis of 218 differentially expressed genes between good responders and poor responders. **c** The top 10 terms identified in the GO enrichment analysis of 218 differentially expressed genes. **d** Venn diagram of the 218 differentially expressed genes and 1811 IRGs from the ImmPort database. **e** Violin plot showing that most of the differentially expressed IRGs were upregulated in good responders. * *p* < 0.05, ** *p* < 0.01, and *** *p* < 0.001

Next, we analysed the differentially expressed genes between good responders and poor responders in the TARGET cohort. Compared to poor responders, 218 differentially expressed genes, including 150 upregulated genes and 68 downregulated genes, were identified in the good responder group (|log FC| ⩾ 0.5, *p* < 0.05) (Fig. 3a). We analysed the potential functions of 218 differentially expressed genes with KEGG and GO enrichment to evaluate the potential functions of the differentially expressed genes. The results of the KEGG pathway analysis revealed that these differentially expressed genes were significantly enriched in immune process or pathway terms, including the TGF-beta signalling pathway and cytokine−cytokine receptor interaction pathway. In addition, several canonical pathways, including the Hippo signalling pathway, PI3K/AKT pathway and Wnt/β-catenin pathway have been reported to play a crucial role in the tumor response to chemotherapy in some cancers, including osteosarcoma [29–31] (Fig. 3b). The GO analysis indicated that these genes correlated with protein binding, DNA-binding transcription factor activity and cell division (Fig. 3c).

ImmPort is a web portal for acquiring IRG lists from NIAID-funded immunology studies, including basic research and clinical trials [32]. Among the set of 218 differentially expressed genes, 24 differentially expressed IRGs were extracted (Fig. 3d). The violin plot showed the upregulation of the expression of most differentially expressed IRGs in good responders (Fig. 3e). These 24 genes intersected and were considered to show predictive power for the chemotherapy response.

### Development of an immune-related signature for predicting the chemotherapy response

**Fig. 4:**
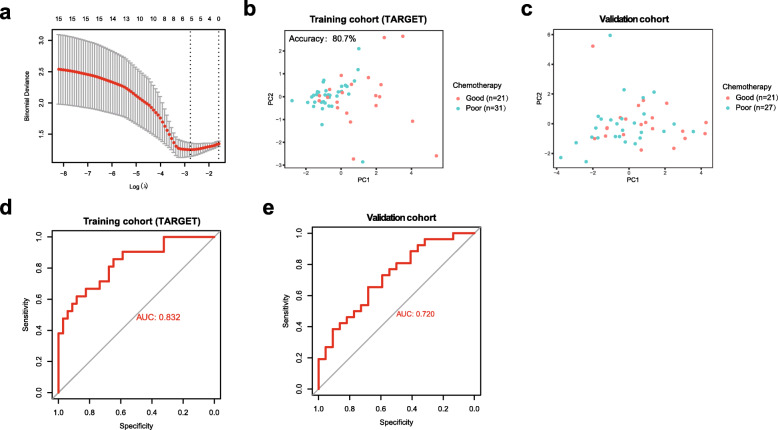
Construction and validation of the immune-related predictive signature. **a** The LASSO method was used to select optimal IRGs for the predictive signature. **b, c** PCA of the predictive signature in the training cohort and validation cohort. **d, e** ROC curve analysis of the predictive signature in the training cohort and validation cohort

We then performed least absolute shrinkage and selection operator (LASSO) logistic regression analysis to develop an IRG signature that would predict the chemotherapy response in patients with osteosarcoma. A binary logistic regression analysis was conducted on 24 differentially expressed IRGs in the training cohort to retrieve the crucial genes that would predict the chemotherapy response. Finally, five IRGs were selected as optimal genes with nonzero regression coefficients (λ) and incorporated into this signature (Fig. 4a). Based on the five genes and their coefficients, the chemotherapy-associated signature was developed in the training cohort. The signature score was calculated using the following formula: CD70*0.0081122515 + TNFRSF9*0.0362462862 + EGFR*0.0561105067 + PDGFD*-0.0081593371 + S100A6*0.000226639702.

Principal component analysis (PCA) showed that the predictive signature drew a clear distinction between good responders and poor responders based on the five immune-related genes in both the training cohort (Fig. 4b) and validation cohort (Fig. 4c). A ROC curve analysis was utilized to evaluate and validate the effect of the predictive signature on the chemotherapy response. The accuracy was 0.807 for the training cohort and 0.689 for the validation cohort. The areas under the curves (AUCs) for the training cohort and validation cohort were 0.832 and 0.720, respectively (Fig. 4d and e). Other signature indexes are shown in Table S3. However, this signature for the prediction of the chemotherapy response did not reach the level of statistical significance in the GSE39055 cohort, likely due to the smaller sample size in this analysis.

**Fig. 5:**
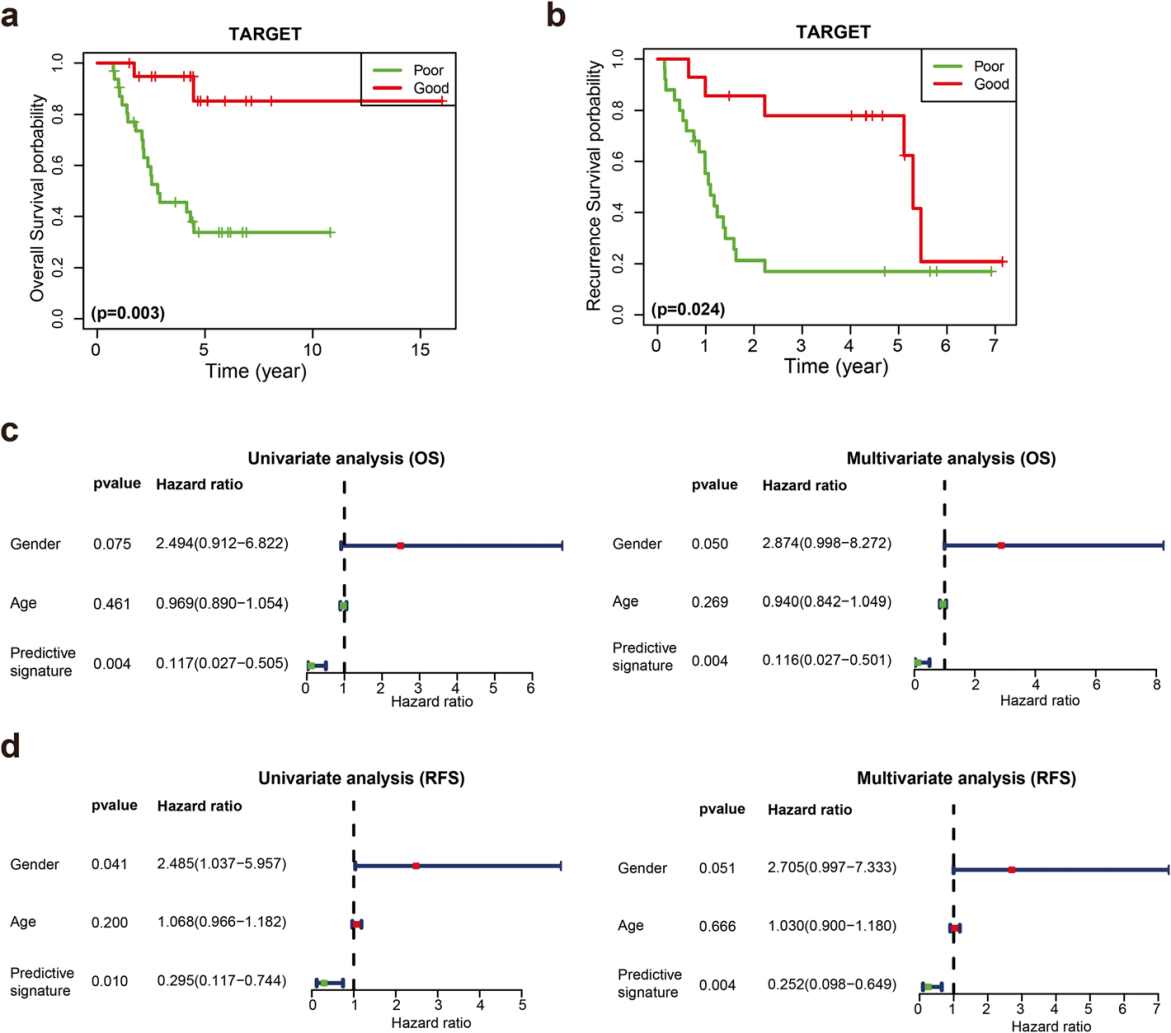
Kaplan-Meier curves for the OS and RFS of good responders and poor responders discriminated by the predictive signature. **a, b** Kaplan-Meier curves for OS and RFS between good responders and poor responders discriminating by predictive signature in the training cohort. **c, d** Forrest plots of the univariate and multivariate analyses showed that the predictive signature for the chemotherapy response predicted OS and RFS in the TARGET cohort independent of clinical factors

Kaplan-Meier curves were generated for the training cohort to evaluate the OS and RFS of good responders and poor responders discriminated by the predictive signature. In the predicted good responder group, patients had longer OS and RFS times than patients in the predicted poor responder group (Fig. 5a and b). We further analysed the correlations of OS and RFS with predictive signature, age and gender by performing univariate and multivariate Cox analyses. Our results indicated that the predictive signature for the chemotherapy response served as an independent prognostic factor for predicting OS and RFS in the training cohort. (Fig. 5c and d).

### Gene set enrichment analysis

**Fig. 6:**
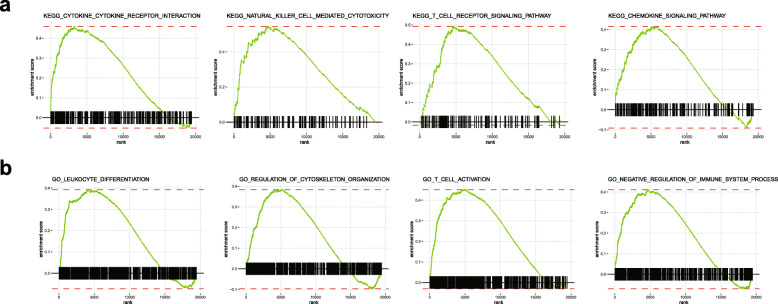
Functional assessment of the immune-related predictive signature using GSEA. **a** The KEGG analysis showed that cytokine-cytokine receptor interaction, natural killer cell-mediated cytotoxicity, T cell receptor signalling pathway and chemokine signalling pathway were increased in the good responder group. **b** The GO analysis showed that leukocyte differentiation, regulation of cytoskeletal organization, T cell activation and negative regulation of immune system processes were increased in the good responder group

We performed a GSEA to investigate the biological processes and signalling pathways between good responders and poor responders in the TARGET cohort based on this predictive signature. The KEGG analysis revealed several immune-related pathways that were significantly enriched in the good responder group, such as cytokine-cytokine receptor interaction, natural killer cell mediated cytotoxicity, T cell receptor signalling pathway and chemokine signalling pathway (Fig. 6a). In the GO analysis, the top 10 GO terms, including leukocyte differentiation, regulation of cytoskeletal organization, T cell activation and negative regulation of immune system process, were increased in the good responder group (Fig. 6b). These results confirmed a positive association between immune cell infiltration and the chemotherapy response in patients with osteosarcoma.

## Discussion

Chemotherapy is an important treatment option that can improve the prognosis of patients with osteosarcoma. High-dose methotrexate, doxorubicin, and cisplatin are the backbones of chemotherapy regimens [33]. However, a significant proportion of patients still do not respond to chemotherapy and experience serious side effects [34]. Therefore, the ability to accurately predict the response to chemotherapy is crucial for the development of appropriate treatments for osteosarcoma. To date, the use of genome-wide expression data to predict the response to chemotherapy in patients with osteosarcoma is still in its infancy. In this study, we developed and validated a five immune-related gene signature for the prediction of the chemotherapy response in patients with osteosarcoma. The identified immune-related predictive signature, including CD70, TNFRSF9, EGFR, PDGFD and S100A6, will facilitate the development of personalized therapy for osteosarcoma and provide new insights into the role of the tumor immune microenvironment in regulating patients’ responses to chemotherapy.

The tumor immune microenvironment is a complex network consisting of immune cells, cytokines and fibroblasts that plays important roles in both cancer treatment and prognosis [35, 36]. In recent years, accumulating evidence has documented the critical role of the immune microenvironment in the determination of the therapeutic response to chemotherapy. Research suggests that tumor-infiltrating immune cells are related to the chemotherapy response in various tumors [20, 37, 38]. For example, Denkert et al. reported that immune cell infiltration was relevant to the response to neoadjuvant chemotherapy and the prognosis after adjuvant chemotherapy in patients with breast cancer [37]. In the present study, we revealed that high-level immune cell infiltration was strongly correlated with a good response to chemotherapy, and CD8 T cells, T follicular helper (Tfh) cells and regulatory T cells were increased in the good responder group. Previous studies have suggested that Tfh cells induce antitumor immunity by activating the effector functions of CD8 T cells [39], and CD8 T cells are the key cell type responsible for the elimination of tumor cells during cancer immunosurveillance [40]. Therefore, the finding that higher levels of CD8 T cells and Tfh cells were strongly associated with a good response was not surprising. Furthermore, chemotherapy has the ability to evoke immunogenic cell death [41, 42], which may partially explain the positive role of CD8 T cells and Tfh cells in the good responder group [43]. In addition, these results suggested that the combination of chemotherapy with immunotherapy may be beneficial for patients with osteosarcoma exhibiting poor response to chemotherapy. Our results revealed that tumor-infiltrating lymphocytes play a crucial role in the tumor response to chemotherapy. Cytotoxic drugs have the potential to increase immunogenicity by triggering immunogenic cell death, which provides therapeutic opportunities for combinations with immunotherapy. Doxorubicin and cyclophosphamide have the powerful capacity to activate immunogenic cell death and are thus attractive for use in combination with immunotherapy [44]. In addition, Wang et al. reported that the combination of an anti-PD-L1 antibody and doxorubicin enhances the antitumor response of the immune system by inhibiting doxorubicin-induced PD-L1 overexpression in osteosarcoma [45].

Recently, gene signatures based on genome-wide expression profiles using genome-wide microarrays or high-throughput sequencing have attracted increasing attention and displayed great potential in predicting chemotherapy responsiveness in various tumors, including breast cancer [46], lung adenocarcinoma [47] and colorectal cancer [17]. With the goal of developing an immune-related gene signature that will identify patients who might benefit from chemotherapy, we analysed gene expression in the TARGET database, where 218 differentially expressed genes were identified between good responders and poor responders. The KEGG analysis indicated that these genes were involved in the TGF-beta signalling pathway and cytokine-cytokine receptor interaction pathway. These results supported the hypothesis that the immune cell infiltration of tumors was associated with the chemotherapy response in patients with osteosarcoma. In addition, the top 10 KEGG categories, including the Hippo signalling and TGF-beta signalling pathways, were also involved in the resistance of various cancer cells to different chemotherapeutic drugs [48, 49].

We deployed a specialized immunological database that may provide insights into the important role of IRGs in the response to chemotherapy to screen potential biomarkers and further explore the role of immune function in chemotherapy. Among these 218 differentially expressed genes, 24 genes overlapped with IRGs from the immunological database. A LASSO regression analysis was applied to retrieve the proportion of intersecting genes and optimize feature selection, which has been broadly used for characteristics of cancer therapy and diagnosis [50], and to facilitate the construction of a practical predictive signature. Finally, a five-gene panel comprising TNFRSF9, CD70, EGFR, PDGFD and S100A6 was included in the predictive signature. Among these five IRGs, TNFRSF9, also known as CD137 or 4-1BB, is an inducible costimulatory receptor known to be expressed on the surface of activated T cells and natural killer (NK) cells. Preclinical results from various tumor models suggested that targeting 4-1BB with agonist antibodies leads to tumor clearance and durable antitumor immunity [51]. In our study, TNFRSF9 was expressed at higher levels in good responders, which may partially explain why these patients are sensitive to chemotherapy. PDGFD is a proangiogenic factor that regulates many cellular processes, including cell proliferation, migration, invasion and angiogenesis [52]. Several studies have reported that PDGFD overexpression is an independent predictor of chemotherapy resistance in ovarian cancer [53] and colorectal carcinoma [54], consistent with our results. In contrast to our expectations, CD70 and S100A6, which were expressed at high levels in good responders in our study, were identified as chemoresistance genes in several studies [55, 56]. In those studies, increased CD70 and S100A6 expression was associated with resistance to chemotherapy and poor survival. Therefore, variability in the response to chemotherapy strongly depends on the genetic and epigenetic profiles of the tumor in these respects.

Subsequently, a chemotherapy predictive signature that consisted of five IRGs was successfully validated as a prediction model in independent GEO datasets. Man et al. and Ochi et al. reported similar results from studies developing a multigene predictive model to classify the chemotherapy response in a few patients with osteosarcoma [57, 58]. Cao et al. and Xiao et al. constructed a multigene prognostic risk model for osteosarcoma. However, no overlapping genes were identified between these gene sets and the 5 IRGs presented in our study. These results indicated a wide range of heterogeneity related to the prognosis and predictive capacity of osteosarcoma. In addition, our study focused on immune cell infiltration and immune-related genes and used different training strategies and algorithms; thus, a comparison of the predictor gene lists among these studies was difficult. We would like to highlight that our study revealed a positive correlation between chemosensitivity and immune cell infiltration in good responders. Immunotherapy has become an established pillar of cancer treatment. The introduction of IRGs may potentially identify new molecular targets for cancer immunotherapy. Therefore, a worthwhile approach would be to evaluate the predictive capacity of immune-related markers as a basis for the identification of new molecular targets for cancer immunotherapy in patients with osteosarcoma.

However, our study had several limitations. First, our study did not include a sufficiently large sample size to comprehensively explore the relationship between IRGs and the chemotherapy response of patients with osteosarcoma. Second, our study was limited by the retrospective nature of the included datasets. Although the five IRG signature has been validated in independent datasets, the prediction of the response to chemotherapy in patients with osteosarcoma has been a more complex issue. Factors such as tumor heterogeneity, the sample size and use of different chemotherapeutic agents that may affect the chemotherapy response of patients with osteosarcoma impede the ability to develop and standardize predictive gene signatures. In this study, the PCA results suggested that the predictive signature seemed to have better predictability for distinguishing patients with a poor response in the training cohort. One explanation for the discrimination is that the sample size is relatively small. To some extent, the discriminatory power depends on the size of the sample. An insufficient sample size has a low probability of detecting a statistically significant difference between treatment groups. Another reason is that tumor heterogeneity may affect the immune-related signature to discriminate between patients with different chemotherapy responses. Therefore, the predictive signature requires further validation in a prospective study with a large sample size.

## Conclusions

We analysed the relevance of immune cell infiltration and gene expression profiles of good responders compared with those of poor responders. We developed a five immune-related gene signature with predictive ability in patients receiving chemotherapy for osteosarcoma, which could be effectively used to predict chemotherapy responsiveness and help improve the efficacy of chemotherapy. Immunotherapy is the next significant breakthrough in cancer treatment. The encouraging results of the present study may provide insights for exploring the molecular mechanisms of chemoresistance and provide a reliable basis for the development of combined immunotherapy approaches for chemotherapy resistance in patients with osteosarcoma.

## Supplementary Information

**Additional file 1: Table S1.** Univariable and multivariable Cox regression analysis of prognostic factors for TARGET cohort. **Table S2.** Univariable and multivariable Cox regression analysis of prognostic factors for GSE39055 cohort. **Table S3.** Performance of immune related signature. **Figure S1.** Immune landscape of the tumor microenvironment between immune infiltration and the chemotherapy response. (a) Unsupervised clustering analysis of patients with osteosarcoma who achieved good and poor responses from the validation cohort and (**b)** GSE39055 cohort using ssGSEA. **Figure S2.** (a) The relationship between immune cell infiltration and the chemotherapy response. Violin plot of good responders and poor responders in the GSE14827 cohort and (**b**) GSE39055, red for good responders and blue for poor responders.

## Data Availability

All data obtained for this study can be found in TARGET (https://ocg.cancer.gov/programs/target). Gene Expression Omnibus (GEO) repository (https://www.ncbi.nlm.nih.gov/geo/) and ImmPort databases (https://www.immport.org/shared/genelists).

## Abbreviations

ssGSEA: Single-sample Gene Set Enrichment analysis
ROC: Receiver operating characteristics
Tfh: T follicular helper cells
FDR: False discovery rate
IRGs: Immune-related genes
LASSO: The least absolute shrinkage and selection operator
PCA: Principal component analysis
AUC: Area under the curve
GSEA: Gene Set Enrichment Analysis
GO: Gene Ontology
KEGG: Kyoto Encyclopedia of Genes and Genomes
RFS: Recurrence free survival
OS: Overall survival
