# Supplementary material for: The tumor immune microenvironment and immune-related signature predict the chemotherapy response in patients with osteosarcoma
Source: BMC Cancer. 2021 May 21;21:581. doi: 10.1186/s12885-021-08328-z (PMC8138974; doi:10.1186/s12885-021-08328-z)
Supplement: Supplementary file 1 — Additional file 1: Table S1. Univariable and multivariable Cox regression analysis of prognostic factors for TARGET cohort. Table S2. Univariable and multivariable Cox regression analysis of prognostic factors for GSE39055 cohort. Table S3. Performance of immune related signature. Figure S1. Immune landscape of the tumor microenvironment between immune infiltration and the chemotherapy response. (a) Unsupervised clustering analysis of patients with osteosarcoma who achieved good and poor responses from the validation cohort and (b) GSE39055 cohort using ssGSEA. Figure S2. (a) The relationship between immune cell infiltration and the chemotherapy response. Violin plot of good responders and poor responders in the GSE14827 cohort and (b) GSE39055, red for good responders and blue for poor responders. [file 12885_2021_8328_MOESM1_ESM.zip › 12885_2021_8328_MOESM1_ESM/Figure S1_ESM.docx]

**Figure S1**


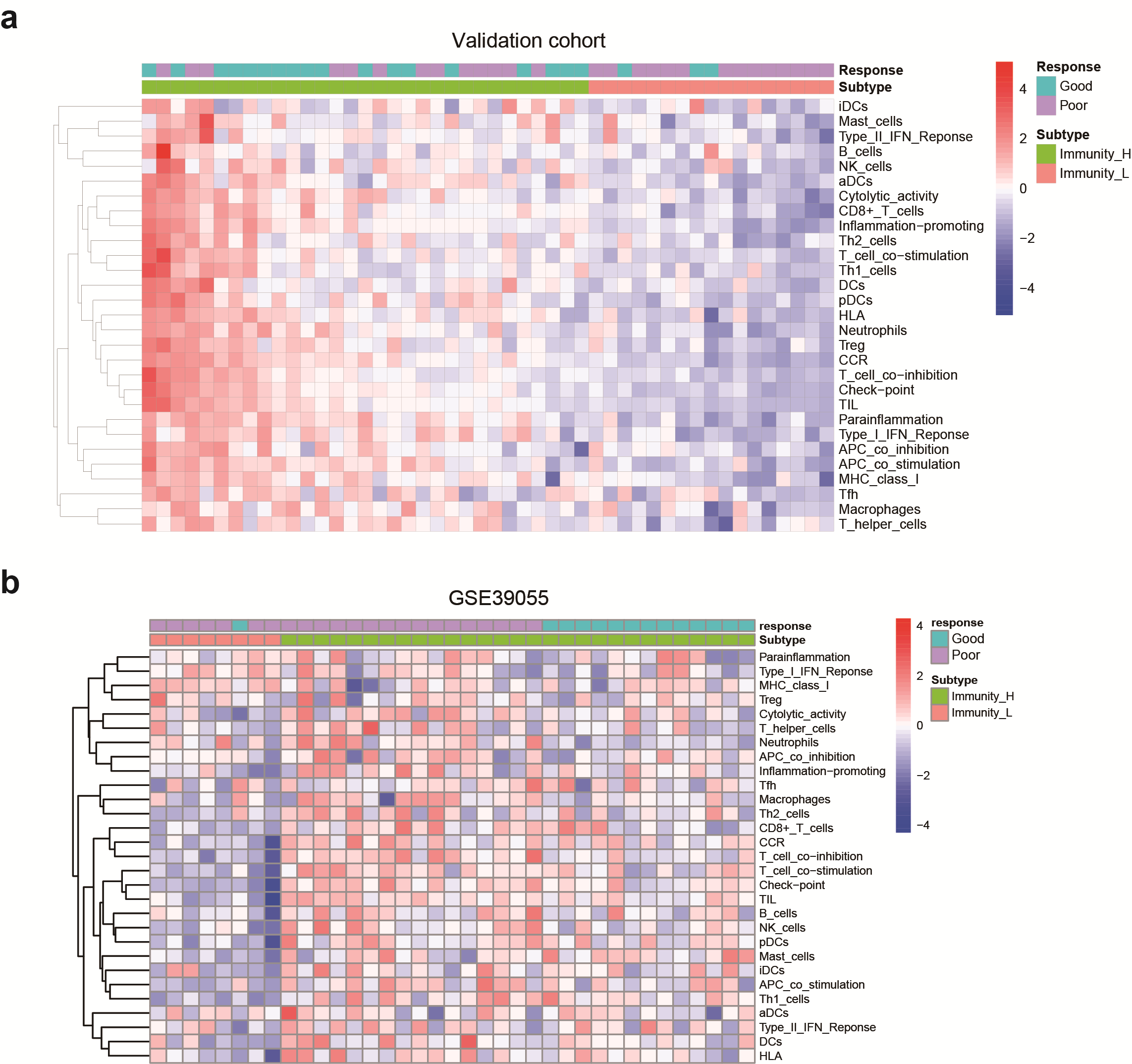


**Figure S1 Immune landscape of the tumor microenvironment between immune infiltration and the chemotherapy response. (a)** Unsupervised clustering analysis of patients with osteosarcoma who achieved good and poor responses from the validation cohort and (**b)** GSE39055 cohort using ssGSEA.
